# Pye’s Surgical Handicraft

**Published:** 1892-09

**Authors:** 


Pye's Surgical Handicraft: A manual of surgical manipulations, minor surgery, and other matters connected with the work of house surgeons and surgical dressers, with three hundred illustrations on wood. First American from the third London edition. Revised and edited by T. H. R. Crowle, F. R. C. S. Complete in one volume. New York: E. B. Treat, No. 5 Cooper Union. 1892. Price, in sheep, $4.00.

This is an admirable volume of six hundred pages, giving the most minute directions for the performance of minor surgical operations.

One of its best features is that the subject of each paragraph is given in small type in the margin of the page. This makes it a handy book of reference. It also has a copious index of twenty-eight pages, which further adds to its value as a book of reference. It deserves a place in every physician's library, and will certainly be found on the shelves of every hospital.

